# Whole-Exome Sequencing Identified a De Novo Mutation of* Junction Plakoglobin* (p.R577C) in a Chinese Patient with Arrhythmogenic Right Ventricular Cardiomyopathy

**DOI:** 10.1155/2019/9103860

**Published:** 2019-05-28

**Authors:** Lv Liu, Chan Chen, YaLi Li, Rong Yu

**Affiliations:** ^1^Department of Respiratory Medicine, Diagnosis and Treatment Center of Respiratory Disease, The Second Xiangya Hospital of Central South University, Changsha, Hunan 410011, China; ^2^Department of Anesthesiology, The Second Xiangya Hospital, Central South University, Hunan 410011, China; ^3^Department of Reproductive Genetics, Hebei General Hospital, Shijiazhuang 050051, China

## Abstract

Arrhythmogenic right ventricular cardiomyopathy (ARVC) is a rare and potentially life-threatening disorder of the heart. The clinical spectrum of ARVC includes myocyte loss and fibro-fatty tissue replacement. With the progress of ARVC, the patient can present serious ventricular arrhythmias, heart failure, and even sudden cardiac death. Previous studies have demonstrated that desmosomes and intermediate junctions play a crucial role in the generation and development of ARVC. In this study, we enrolled a Chinese patient with suspicious ARVC. The patient suffered from right ventricular enlargement and less thickening of right ventricular wall. ECG record showed an epsilon wave. However, there was no obvious symptom in his parents. After whole-exome sequencing and data filtering, we identified a de novo mutation (c.1729C>T/p.R577C) of* junction plakoglobin* (*JUP*) in this patient. Bioinformatics programs predicted that this mutation was deleterious. Western blot revealed that, compared to cells transfected with WT plasmids, the expressions of* desmoglein 2* (*DSG2*) and* Connexin 43 *were decreased overtly in cells transfected with the mutant plasmid. Previous studies have proven that the reduction of* DSG2* and* Connexin 43* may disturb the stability of desmosomes. In this research, we reported a novel de novo mutation (c.1729C>T/p.R577C) of* JUP* in a Chinese patient with suspicious ARVC. Functional research further confirmed the pathogenicity of this novel mutation. Our study expanded the spectrum of* JUP* mutations and may contribute to the genetic diagnosis and counseling of patients with ARVC.

## 1. Introduction

Arrhythmogenic right ventricular cardiomyopathy (ARVC, #107970) is a hereditary desmosomal disorder with right ventricular and left ventricular dysfunction [1, 2]. It is characterized by cardiomyocytes loss and fibro-fatty tissue replacement. However, some patients also present atypical phenotypes such as mimicking hypertrophic or dilated cardiomyopathy affecting the left ventricle without overt evidence of the pathognomonic fatty fibrous replacement [3, 4].

An epidemiological survey showed that the prevalence of ARVC was more than 0.02% worldwide [5]. It is a crucial underlying cause of ventricular arrhythmias, heart failure, and sudden cardiac death (SCD). At present, mutations in more than ten genes like* plakophilin 2* (*PKP2*),* desmoplakin* (*DSP*),* desmocollin 2* (*DSC2*),* desmoglein 2* (*DSG2*), and* junction plakoglobin* (*JUP*) may be responsible for ARVC as have been reported [6, 7].

In this study, we employed whole-exome sequencing to explore the candidate gene in a patient with suspicious ARVC. In combination with bioinformatics analysis and Sanger sequencing validation, a de novo mutation (c.1729C>T/p.R577C) of* JUP* was identified as the underlying genetic lesion of this patient. Western Blot research confirmed that this mutation may affect the expression of DSG2 and Connexin 43 and finally disturb the stability of desmosome junction.

## 2. Materials and Methods 

### 2.1. Subjects

**Figure 1:**
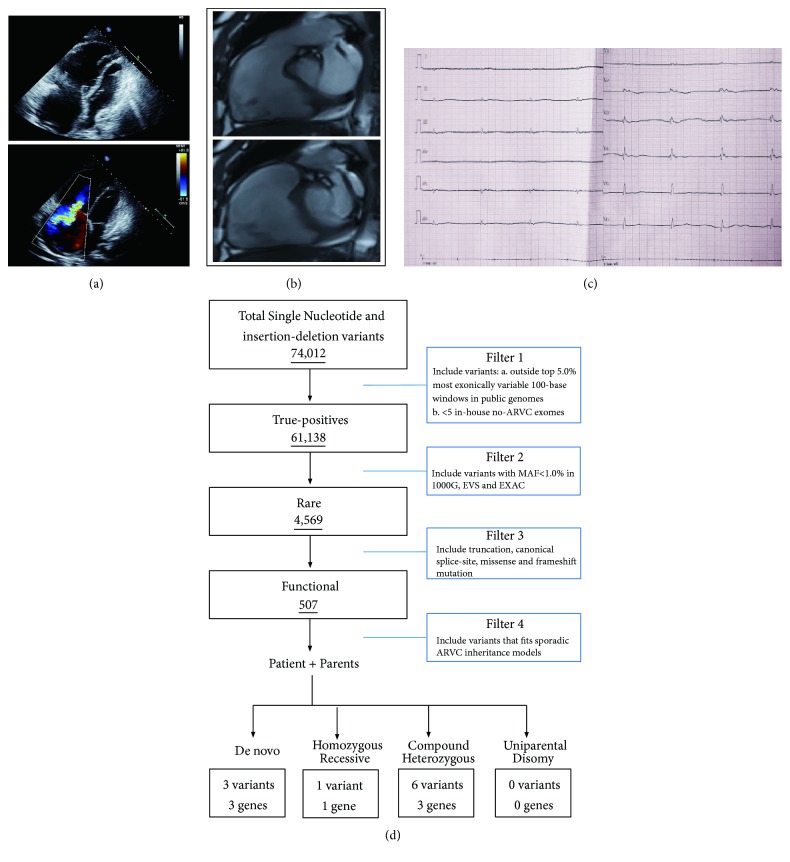
The clinic data of the patient. (a) The B ultrasonic testing of the patient. (b) The MRI testing of the patient. (c) The EGC testing data of the patient. (d) The strategies of whole-exome sequencing data filtering.

In this study, we enrolled a patient with suspicious ARVC from the central south of China. The proband, a twenty-four-year-old male from Hunan province, was admitted to our hospital due to syncope during sports class. B ultrasonic testing and Magnetic Resonance Imaging (MRI) all indicated right ventricular enlargement (LV=63 mm) and less thickening of right ventricular wall (Figures 1(a) and 1(b)). Electrocardiogram (ECG) testing showed epsilon wave (T-wave inversion) (Figure 1(c)). So, this patient was diagnosed as suspicious ARVC. But his parents did not show any symptom. All participants gave written informed consent.

### 2.2. Whole-Exome Sequencing

Genomic DNA was extracted from peripheral blood lymphocytes of all the family members with a DNeasy Blood & Tissue Kit (Qiagen, Valencia, CA) following the manufacturer's instruction [8]. The central part of whole-exome sequencing was provided by the Novogene Bioinformatics Institute (Beijing, China). The exomes were captured using Agilent SureSelect Human All Exon V6 kits, and the platform of high-throughput sequencing was performed in Illumina HiSeq X-10. The necessary bioinformatics analysis, including reads, mapping, variant detection, filtering, and annotation, was also endowed by Novogene Bioinformatics Institute as we previously described [9, 10]. The strategies of data filtering referred to Figure 1(d).

### 2.3. Mutation Validation and Cosegregation Analysis

All the filtered mutations of this family were validated by Sanger sequencing. The primer pairs (the sequence of primers will be provided upon request) were designed by Primer 5. The sequences of the polymerase chain reaction (PCR) products were determined using the ABI 3100 Genetic Analyzer [11] (ABI, Foster City, CA).

### 2.4. Cell Culture

AC16 cardiomyocytes were cultured in Dulbecco's Modified Eagle's Medium supplemented with 10% (v/v) fetal bovine serum in an incubator at 37°C and 95% CO_2_. The cell culture media was changed every 2 days. The cells were seeded at an appropriate density according to each experimental design.

### 2.5. Mutagenesis and Cell Transfection

We designed a wild-type JUP CDS plasmid with HIS-tag in a pcDNA3.1+ vector. The R577C-JUP missense mutation was engineered into the vector using the Takara MutanBEST Kit (Takara Bio, Otsu, Shiga, Japan). AC16 cells were transfected with HIS-JUP-pcDNA3.1+ (WT and p.R577C) by using Lipofectamine™ 2000 CD Transfection Reagent (Thermo Fisher Scientific), following the manufacturer's instructions.

### 2.6. Western Blot

For Western blot analysis, the cultured AC16 cells were homogenized on ice in 1% CHAPS extraction buffer (150 mM KCl, 50 mM HEPES, pH 7.4, 0.1% CHAPS) containing complete™ EDTA-free Protease Inhibitor (Roche Bioscience) and 0.1 mM Na_3_VO_4_ to inhibit phosphatases. The homogenates were rotated for 30 min at 4°C to ensure the extraction of membrane proteins. After centrifugation at 15,000 × g for 120 min, the supernatant was collected, and protein concentrations were measured with BCA protein assay reagent (Pierce). Equal amounts of lysate proteins were resolved on 4–12% Bis-Tris NuPAGE gels, followed by standard Western blotting with the antibodies specified below. Chemiluminescent signals were scanned, and integrated density values were calculated with a chemiluminescent imaging system (Alpha Innotech). The antibodies of HIS, DSG2, Connexin 43, and GAPDH were purchased from Cell Signaling Technology (USA).

## 3. Results

### 3.1. Whole-Exome Sequencing Identified a De Novo Mutation in JUP

The DNA paternity testing results showed that all 15 STK loci and one sex recognition locus of the proband were all consistent between both parents, which indicated that the parents were biologically related to the child, and the probability of parentage was 99.99%.

**Figure 2:**
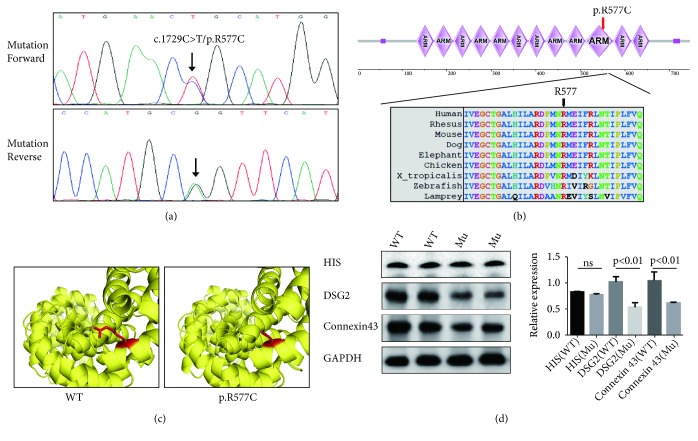
The genetic analysis and functional research of the p.R577C mutation. (a) Sequencing results of the* JUP* mutation. Sequence chromatogram indicates a C to T transition of nucleotide 1729. (b) Analysis of the mutation and protein domains of JUP. The R577 affected amino acid locates in the highly conserved amino acid region in different mammals (from Ensembl). The black arrow shows the R577 site. (c) Swiss-model analyzed the JUP structures of WT and Mutated (p.R577C). (d) Western blot analyzed the levels of HIS, DSG2, Connexin 43, and GAPDH in normal and mutation cells lysis. *∗∗∗* represents p< 0.001.

Whole-exome sequencing yielded 9.16 Gb data with 99.2% coverage of the target region and 98.4% of target covered over 10×. In total, 74,012 variants were present in the proband. We then performed the data filtering as Figure 1(d). The filtered data were shown in Table 1. We have identified 10 mutations in 7 genes finally. The mutations of* MEF2A*,* IGFN1*,* DNAH6*, and* ITPR3* were excluded by OMIM phenotypes. Bioinformatics analysis found that the expressions of* DCST1* and* NPIPB6* were lower than JUP according to GTEx [12]. And ToppGene [13] Function analysis also suggests that JUP mutation has a high possibility to be a causative factor. Bioinformatics analysis including GTEx, ToppCluster, SIFT [14], PolyPhen-2 [15], and MutationTaster [16] predicted a de novo mutation (c.1729C>T/p.R577C) of* JUP* as the underlying genetic lesion of this patient (Figure 2(a)). This novel mutation, resulting in a substitution of arginine by cysteine at codon 577 in exon 10 of* JUP* gene, was also not found in our 200 local control cohorts [9], 1000G, dbSNP, Exome Variant Server database (http://evs.gs.washington.edu/EVS/), and Chinese control population (YH database). The frequency of the mutation in the EXAC database is 0.0000579; in gnomAD it is 0.0000365; all the MAF were far less than 0.01. The cross-species alignment analysis of JUP amino acid sequences revealed that this mutated site was highly evolutionarily conserved (Figure 2(b)).

### 3.2. The De Novo Mutation May Affect the Expression of DSG2 and Connexin 43 and Disturb the Stability of Desmosome Junction

We also constructed the molecular structure model by Swiss-model (Figure 2(c)). And the SDM software predicted that this novel mutation may reduce the stability of this protein based on the molecular structure model we have constructed [17].

We then designed the HIS-JUP-pcDNA3.1+ (WT and p.R577C) plasmids and transfected them to do Western blot. Western blot showed that the expression of DSG2 and Connexin 43 [18, 19] in mutated cells decreased (Figure 2(d)). The expression of JUP-HIS had no obvious change. These data indicated that the novel mutation (p.R577C) disturbed the stability of desmosome junction

## 4. Discussion

In this study, we enrolled a patient with suspicious ARVC; whole-exome sequencing identified a de novo mutation (c.1729C>T/p.R577C) of* JUP* in the patient. Functional studies further confirmed that this mutation may affect the expressions of DSG2 and Connexin 43 and disturb the stability of desmosome junction. In Clinvar database (https://www.ncbi.nlm.nih.gov/clinvar/variation/468747/), this mutation (p.R577C) of* JUP* was included, but this mutation is described as “uncertain significance” in Clinvar and was not included in HGMD (the Human Gene Mutation Database) and no paper reported this mutation. So, we confirmed the pathogenicity of the* JUP* mutation (p.R577C) by whole-exome sequencing and functional research in this study.

The human* JUP* gene encoding junction plakoglobin protein is located on chromosome 17q21.2, and it consists of 14 exons, spanning approximately 42 kilobases (kb). The human JUP can be divided into three regions: the N-terminal, C- terminal, and 12 ARM repeats domain [19, 20] (Figure 2(b)). In previous studies, most of* JUP* mutations were identified in ARVC patients [21, 22]. In our study, we also identified a novel* JUP* mutation (c.1729C>T/p.R577C) in a suspected ARVC patient. The mutation (p.R577C) was located in the tenth ARM repeat domain; previous research has demonstrated that this conserved domain played a crucial role in desmosomes. The site from 574aa to 661aa can bind with DSG1 to maintain the stability of desmosomes and intermediate junctions [23, 24]. Our study implied that this mutation (p.R577C) may disturb the stability of desmosomes and intermediate junctions and lead to diseases in the end.

To date, 41 mutations of* JUP* have been identified in patients with ARVC and cutaneous problem. And, in the tenth ARM domain, one mutation (p.Q539X) was reported to cause epidermolysis bullosa [25], and one mutation (p.V603L) was identified in ARVC patient [21]. This difference may be caused by different types of mutation as well as incomplete penetrance. So, it is difficult to confirm the diagnosis of ARVC based on the general clinical examination because of the nonspecific nature of the disease and the broad spectrum of phenotypic variations [26, 27]. The genetic analysis and sequencing may make some contributions to the diagnosis of ARVC. And, in our study, the patient shows no obvious cutaneous problem. Certainly, we will perform a follow-up of this patient to do further investigation [28].

## 5. Conclusion

In conclusion, via whole-exome sequencing in the combination of bioinformatics analysis strategy, we have identified a de novo* JUP* mutation (c.1729C>T/p.R577C) in a suspected ARVC patient from central south of China. Functional research implied that this novel mutation may disturb the stability of desmosomes and intermediate junctions. Our study may expand the spectrum of* JUP* mutations and contribute to genetic diagnosis and counseling of ARVC.

## Figures and Tables

**Table 1:** The filtered data of whole exome sequencing.

Gene	Inheritance		Transcript Variant	Protein Variant	SIFT	PolyPhen-2	MutationTaster	OMIM Clinical Phenotype	GTEx (expression in heart)	ToppGene Function
*JUP*	De novo	-* *-	c.1729C>T	p.R577C	Damaging	Damaging	Disease-causing	AD, Arrhythmogenic right ventricular dysplasia	84.83	Desmosome assembly
*MEF2A*	De novo	-* *-	c.335C>T	p.P112L	Tolerated	Damaging	Disease-causing	AD, Coronary artery disease	32.23	Mitochondrion distribution; Cardiac myofibril assembly
*DCST1*	De novo	-* *-	c.1004delG	p.R335fs	Damaging	Damaging	Disease-causing	-* *-	0.7	Antigen processing and display for immune responses
*NPIPB6*	HR	Paternal	c.983C>G	p.P328R	Damaging	Damaging	Disease-causing	-* *-	0.12	-* *-
		Maternal								
*IGFN1*	CH	Paternal	c.86C>T	p.P29L	Damaging	Damaging	Disease-causing	-* *-	0.3	Contractile fiber part
		Maternal	c.1253A>C	p.Q418P	Tolerated	Unknown	Polymorphism			
*DNAH6*	CH	Paternal	c.2912G>A	p.R971K	Damaging	Damaging	Polymorphism	AR, Primary ciliary dyskinesia	0.08	Cilium movement; Microtubule-based movement
		Maternal	c.3458G>A	p.R1153Q	Tolerated	Damaging	Disease-causing			
*ITPR3*	CH	Paternal	c.1132G>A	p.D378N	Tolerated	Damaging	Disease-causing	AR, Diabetes	11.23	Inositol phosphate-mediated signaling
		Maternal	c.4185C>G	p.D1395E	Damaging	Benign	Disease-causing			

Transcript IDs: JUP, NM_002230; MEF2A, NM_001130928; DCST1, NM_152494; NPIPB6, NM_001282524; IGFN1, NM_001164586; DNAH6, NM_001370; ITPR3, NM_002224. AD, autosomal dominant; AR, autosomal recessive; CH, compound heterozygous; HR, homozygous recessive; OMIM, Online Mendelian Inheritance in Man.

## Data Availability

The data used to support the findings of this study are available from the corresponding author upon request.
